# Editorial: Climate change challenge in pediatric psychology

**DOI:** 10.3389/fpsyg.2024.1439041

**Published:** 2024-06-14

**Authors:** Livio Provenzi, Lucia Billeci, Caradee Wright, Zhiwei Xu

**Affiliations:** ^1^Department of Brain and Behavioral Neuroscience, University of Pavia, Pavia, Italy; ^2^Developmental Psychobiology Lab, IRCCS Mondino Foundation, Pavia, Italy; ^3^Institute of Clinical Physiology, National Research Council (CNR-IFC), Pisa, Italy; ^4^Department of Clinical and Experimental Medicine, University of Pisa, Pisa, Italy; ^5^Climate Change and Health Research Programme, Environment and Health Research Unit, South African Medical Research Council, Pretoria, South Africa; ^6^Department of Geography, Geoinformatics and Meteorology, University of Pretoria, Pretoria, South Africa; ^7^School of Medicine and Dentistry, Griffith University, Gold Coast, QLD, Australia

**Keywords:** climate change, eco-anxiety, climate anxiety, education, pollution

## A looming storm: climate change and child development

The United Nations Sustainable Development Goals (SDGs) aim to develop healthy societies aligned with collective wellbeing (Saxena et al., 2021). Goal 13 on Climate Action specifically calls for urgent action to combat climate change and its impacts. The impact of climate change on children's mental health has been a growing concern in recent years. Studies have shown that exposure to climate-related disasters can lead to increased rates of post-traumatic stress disorder, anxiety, and depression in children (Sharpe and Davison, 2021; Rothschild and Haase, 2023). Furthermore, not only the direct exposure to polluted environments (Shezi and Wright, 2018; Nazzari et al., 2023; Pili et al., 2024), but also the chronic stress associated with the uncertainty and fear surrounding climate change can have long-lasting effects on children's emotional development and resilience (Léger-Goodes et al., 2022). While the consequences of this environmental crisis are wide-ranging, one area often overlooked is its profound impact on the mental health of our children. This Research Topic dives headfirst into this critical issue, exploring the complex interplay between climate change and the emotional wellbeing of our youth. By contributing to achieving SDG 13, this Research Topic also offers unique insights into different aspects and dimensions related to climate change challenge for pediatric psychology.

As Newberry Le Vay et al. point out in their paper, climate change is the greatest threat humanity faces, and its effects are already being felt by children and young people around the world. They argue for a paradigm shift in climate change education, one that goes beyond simply imparting knowledge about our warming planet. They specifically suggest integrating mental health considerations into a “whole school approach” curriculum. By equipping children with not just scientific understanding but also the emotional resilience to navigate a future fraught with uncertainty, we empower them to become not just informed citizens, but active participants in building a more sustainable world. It is noteworthy that for such educational mission to be successful, a virtuous exchange and collaborative efforts of multiple stakeholders and experts—from researchers to educators, from clinicians to citizens—is critically needed in order to deal with systemic barriers in climate change education.

However, as Nadarajah et al. remind us, a one-size-fits-all approach will not suffice. Their study delves into the social factors that shape young people's perceptions of climate change. They highlight the influence of social background and scientific interest, emphasizing that students from lower socioeconomic backgrounds might have limited awareness of the social and economic ramifications of climate change. Moreover, they also highlight the relevance of educating the entire citizenship on the ecological transition and its implications. This underscores the need for educational programs that cater to diverse learning styles and backgrounds, ensuring that no child is left behind in understanding the gravity of the situation. At the same time, as highlighted further in this editorial, education should come together with adequate provision of psychological resources and tools to deal with potential triggers of eco-anxiety.

The qualitative research presented by Schürr et al. provides a stark reminder of the immediate mental health consequences of extreme weather events (EWEs), a phenomenon expected to become more frequent and intense with climate change. Their study explores the lived experience of adolescents living in a German town affected by flooding in 2016. The interviews showed the lingering anxieties, feelings of helplessness, and disruptions to daily life that can follow such disasters. The need of psychological support was not only heightened during the immediate aftermath; rather, it was a continuous urgency in the long run. Critically, their findings emphasize the importance of accessible and long-term mental health support systems for young people who have experienced these traumatic events. Early intervention and readily available resources can make a significant difference in mitigating the long-term psychological impact of climate-related disasters.

La Greca et al. contribute to this discussion by focusing on the often-overlooked physical manifestations of the emerging phenomenon of climate anxiety. Their study examines the link between evacuation stress during Hurricane Irma hit in Florida and the development of physical health problems in children. Their findings reveal that the emotional strain associated with potential disaster events, even without direct exposure, can lead to somatic complaints in young people. This highlights the need for comprehensive disaster preparedness plans that go beyond physical safety considerations and incorporate strategies for managing stress and anxiety in both children and adults.

## To glimpse a landscape

By adopting a multi-layer approach, we can begin to mitigate the negative impacts of climate change on our children's mental health. Key effective actions to target the climate change challenge in pediatric psychology highlighted by the present Research Topic include, but are not limited to:

- Empowering education: integrate mental health considerations into climate change education, equipping children with the knowledge, coping mechanisms, and emotional resilience to navigate a changing world.
- Bridging the gap: address social inequalities in climate change education, ensuring that all children, regardless of socio-economic standing, have access to comprehensive and engaging programs that foster environmental literacy and a sense of agency.
- Building resilience: foster a culture of resilience in our communities by teaching children healthy coping mechanisms for dealing with stress and anxiety, particularly in the face of climate-related disasters.
- Accessible mental health care: invest in accessible and affordable mental health care services for children and adolescents, ensuring that those struggling with climate anxiety or trauma have the support they need.
- Open communication: create an open and honest dialogue about climate change with children, fostering a sense of shared responsibility and empowering them to participate in solutions.

By prioritizing our children's mental wellbeing, we not only ensure they are equipped to face the challenges of a changing climate, but we also cultivate a generation of informed and empowered citizens who will be at the forefront of building a more sustainable future (Chersich et al., 2019; Roos et al., 2021). The storm may be looming, but by working together, we can provide our children with the tools they need to weather it. And we need to start glimpsing the landscape today by embracing a preventive and nurturing approach.

## Establishing community hubs for building a cultural and educational support system

In addition to the points mentioned above, we can also explore the role of communities in supporting children's mental health (Xu et al., 2012; Monroe et al., 2019). Schools, youth groups, and environmental organizations can all play a vital role in providing safe spaces for children to discuss their anxieties and concerns about climate change. Trained educators and facilitators can guide these conversations, fostering a sense of community and shared experience (Rousell and Cutter-Mackenzie-Knowles, 2020; Kumar et al., 2023). Furthermore, incorporating mental health preventive practices, relaxation techniques, and social-emotional learning programs into school curriculums can equip children with the tools they need to manage stress and anxiety (Malboeuf-Hurtubise et al., 2024).

Fostering a sense of connection to nature can be another critical tool for building resilience in educational settings (Sobko et al., 2018). Encouraging outdoor exploration, nature-based activities, and environmental stewardship can help children develop a deeper appreciation for the natural world and a stronger commitment to protecting it (Pérez-del-Pulgar et al., 2021). Studies have shown that spending time in nature can reduce stress, improve mood, and boost cognitive function (Ye et al., 2024) even in subjects with neuropsychiatric disorders (Curzio et al., 2022). Connecting children with the natural world can not only improve their mental health but also empower them to become active participants in finding solutions to the climate crisis.

Feeling helpless in the face of a massive global challenge like climate change can be paralyzing. However, empowering children to act, even in small ways, can foster a sense of agency and hope. Schools and communities can create opportunities for children to participate in environmental projects, such as planting trees, cleaning up local waterways, or advocating for sustainable practices. By giving children a voice and a platform to contribute to positive change, we can help them feel empowered to be part of the solution (Chou et al., 2023; Léger-Goodes et al., 2023).

## Conclusions

The mental health impacts of climate change on children are a complex and growing concern. However, by acknowledging the problem, taking action, and working together, we can build a future where children are not only informed about the climate change challenge we face today, but also equipped with the emotional resilience and sense of agency they need to navigate an ever-changing world. By prioritizing their mental wellbeing, we invest not just in the health of our children but also in the future of our planet. With open communication, sustainable commitment to equity in education and mental health support, and a focus on building a culture of resilience and action, we can empower our children to weather it and build a brighter future for all.

## Author contributions

LP: Writing – review & editing, Writing – original draft. LB: Writing – review & editing, Writing – original draft. CW: Writing – review & editing, Writing – original draft. ZX: Writing – review & editing, Writing – original draft.
